# Probing delivery of a lipid nanoparticle encapsulated self-amplifying mRNA vaccine using coherent Raman microscopy and multiphoton imaging

**DOI:** 10.1038/s41598-024-54697-3

**Published:** 2024-02-22

**Authors:** Kajari Bera, Renán A. Rojas-Gómez, Prabuddha Mukherjee, Corey E. Snyder, Edita Aksamitiene, Aneesh Alex, Darold R. Spillman, Marina Marjanovic, Ahmed Shabana, Russell Johnson, Steve R. Hood, Stephen A. Boppart

**Affiliations:** 1 GSK Center for Optical Molecular Imaging, University of Illinois Urbana-Champaign, Urbana, IL USA; 2 Beckman Institute for Advanced Science and Technology, University of Illinois Urbana-Champaign, Urbana, IL USA; 3 Department of Electrical and Computer Engineering, University of Illinois Urbana-Champaign, Urbana, IL USA; 4 In Vitro/In Vivo Translation, Research, GlaxoSmithKline, Collegeville, PA USA; 5 Department of Bioengineering, University of Illinois Urbana-Champaign, Urbana, IL USA; 6 Carle Illinois College of Medicine, University of Illinois Urbana-Champaign, Urbana, IL USA; 7 GSK Vaccines, Rockville Center for Vaccines Research, Rockville, MD USA; 8 In Vitro/In Vivo Translation, Research, GlaxoSmithKline, Stevenage, UK; 9 Cancer Center at Illinois, University of Illinois Urbana-Champaign, Urbana, IL USA

**Keywords:** Self-amplifying mRNA, CARS, FLIM, Lipid nanoparticles, Imaging, Fluorescence imaging, Imaging and sensing

## Abstract

The COVID-19 pandemic triggered the resurgence of synthetic RNA vaccine platforms allowing rapid, scalable, low-cost manufacturing, and safe administration of therapeutic vaccines. Self-amplifying mRNA (SAM), which self-replicates upon delivery into the cellular cytoplasm, leads to a strong and sustained immune response. Such mRNAs are encapsulated within lipid nanoparticles (LNPs) that act as a vehicle for delivery to the cell cytoplasm. A better understanding of LNP-mediated SAM uptake and release mechanisms in different types of cells is critical for designing effective vaccines. Here, we investigated the cellular uptake of a SAM-LNP formulation and subsequent intracellular expression of SAM in baby hamster kidney (BHK-21) cells using hyperspectral coherent anti-Stokes Raman scattering (HS-CARS) microscopy and multiphoton-excited fluorescence lifetime imaging microscopy (FLIM). Cell classification pipelines based on HS-CARS and FLIM features were developed to obtain insights on spectral and metabolic changes associated with SAM-LNPs uptake. We observed elevated lipid intensities with the HS-CARS modality in cells treated with LNPs versus PBS-treated cells, and simultaneous fluorescence images revealed SAM expression inside BHK-21 cell nuclei and cytoplasm within 5 h of treatment. In a separate experiment, we observed a strong correlation between the SAM expression and mean fluorescence lifetime of the bound NAD(P)H population. This work demonstrates the ability and significance of multimodal optical imaging techniques to assess the cellular uptake of SAM-LNPs and the subsequent changes occurring in the cellular microenvironment following the vaccine expression.

## Introduction

Vaccines are the foundation of public health programs, have major socioeconomic benefits, and have saved millions of lives globally^1^. As is evident from the rapid development of RNA vaccines during the global SARS-CoV-2 pandemic, there has been a shift in vaccinology toward synthetic RNA platforms: conventional messenger ribonucleic acid (mRNA) and self-amplifying mRNA (SAM) vaccines^2^. Traditional platforms such as live-attenuated or inactivated vaccines are limited by the requirement for intricate cell culture technologies, lengthy safety assessments, and difficulties in production scale-up^3^. In comparison, mRNA vaccine platforms allow for rapid, scalable, and cell-free manufacturing of prophylactic and therapeutic vaccines.

SAM is a synthetic mRNA vaccine platform that encodes the antigen of interest and the viral replication machinery required for intracellular RNA amplification^4^. Unlike conventional mRNA vaccines, SAM can generate many copies of the mRNA in the target cell, leading to high and prolonged expression of the antigen, and can elicit protective immune responses at lower doses^5,6^. For an mRNA vaccine to be efficacious, mRNA molecules have to reach target cells and produce sufficient antigens of interest. Large doses or repeat administrations may be needed to achieve adequate protection. Since mRNA is highly unstable under physiological conditions, safe and stable delivery systems that protect them from degradation, allow cellular uptake, and intracellular mRNA release are required. Lipid nanoparticles (LNPs) are one of the most advanced delivery systems for mRNA vaccines^7^. Notably, two of the Food and Drug Administration (FDA) approved SARS-CoV-2 vaccines, mRNA-1273 and BNT162b, use LNPs to deliver mRNA^8,9^.

New imaging technologies capable of visualizing and evaluating the cellular uptake process of vaccine formulations and their immunogenic properties are essential for developing safe and effective next-generation vaccine platforms. In the past two decades, significant advancements have been made in the field of nonlinear optical imaging technologies capable of providing high-resolution structural as well as functional information of biological samples using label-based and label-free approaches non-invasively and in vitro^10–15^. Compared to conventional brightfield and confocal imaging techniques, nonlinear optical imaging techniques such as multiphoton-excited fluorescence (MPEF) intensity, multiphoton-excited fluorescence lifetime imaging microscopy (FLIM), and simultaneous label-free autofluorescence multiharmonic (SLAM) microscopy provide several benefits for live cell imaging studies, such as higher signal-to-noise ratio (SNR), lower phototoxicity, and increased depth of penetration into the samples^16–21^. Multiphoton FLIM, which is an extension of MPEF and offers the ability to probe the cellular metabolic state, is sensitive to changes in the cellular micro-environment based on fluorescence lifetime measurements.

Coherent anti-Stokes Raman scattering (CARS) microscopy is another label-free multiphoton imaging technique that permits non-destructive chemical imaging of live cells based on the intrinsic vibrational contrast of its molecules^22–25^. CARS microscopy has been widely used to visualize lipid bilayers, cell membranes, and lipid droplets without any labelling^26–30^. This advantage is important for imaging small molecules such as lipids where labelling may significantly affect their molecular properties. Among the various technical implementations of CARS microscopy, the hyperspectral CARS (HS-CARS) imaging approach in which a CARS spectrum is collected at each spatial location provides superior chemical specificity compared to single-frequency CARS^26,30^. In this study, we investigated the capability of multimodal multiphoton imaging techniques such as MPEF and HS-CARS to image and quantify the cellular uptake of SAM-LNP formulations in vitro. Additionally, multiphoton-excited FLIM was utilized for characterizing the functional changes occurring in the cellular microenvironment following uptake of the SAM-LNP vaccine in baby hamster kidney (BHK-21) cells.

## Results and discussion

### Visualization of SAM-GFP-LNPs cellular uptake and expression kinetics in vitro

The assessment of a drug metabolism and its kinetics in vitro and in vivo is critical in pharmaceutical research. Herein we investigated the cellular uptake and expression kinetics of a SAM vaccine encoding green fluorescent protein (GFP). The SAM vaccine served as a model antigen of interest and was delivered by LNPs. In this paper, the vaccine formulation is referred to as SAM-GFP-LNPs. Encapsulation of macromolecules in a LNP is known to protect the therapeutic agent during transport through the body and facilitate the intracellular delivery via a fusion-based pathway^31^. GFP expression was used as the marker to evaluate SAM-GFP-LNPs uptake kinetics and expression patterns in vitro^32^.

**Figure 1:**
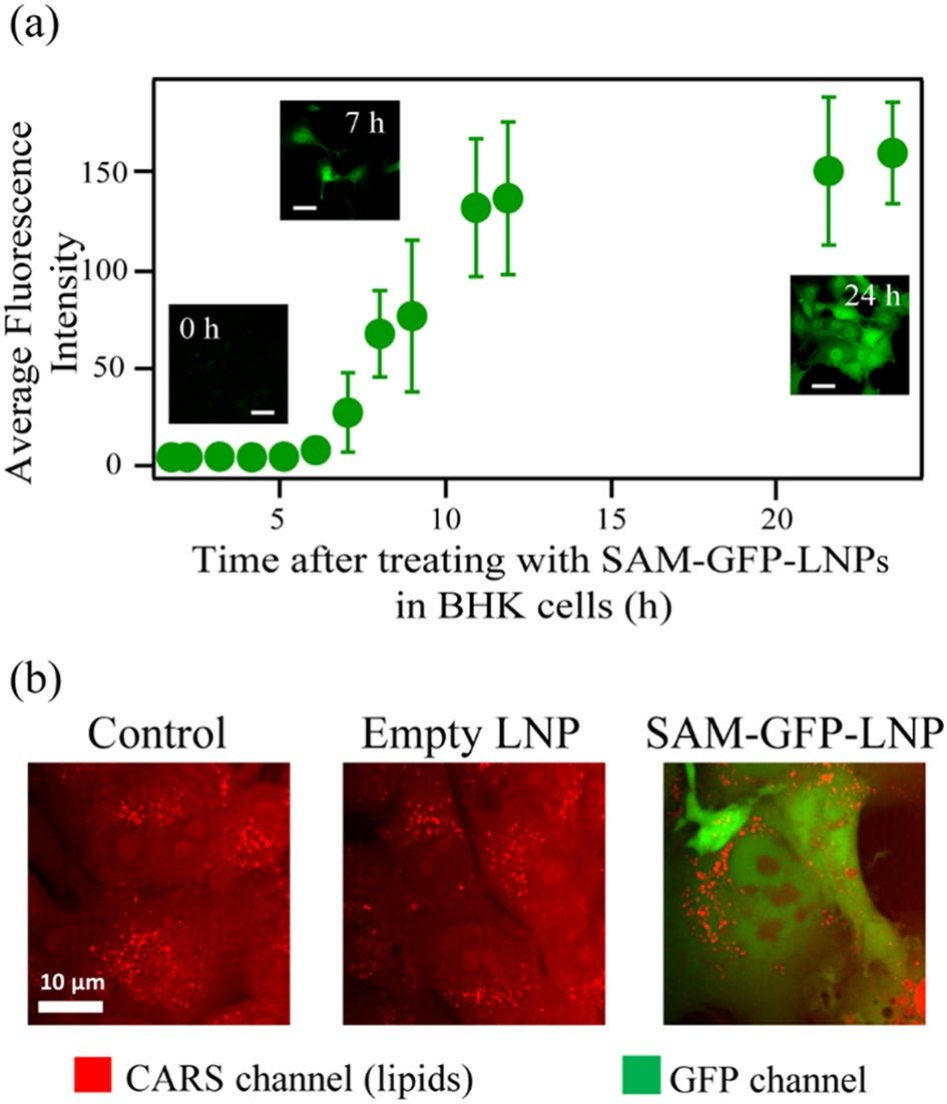
Kinetics and spatial localization of SAM-GFP-LNPs. (**a**) Green fluorescence protein (GFP) signal kinetics in BHK-21 cells treated with the SAM-GFP-LNPs formulation. Scale bars correspond to 45 µm. (**b**) Representative simultaneous CARS/MPEF images of BHK-21 cells from control, empty LNP, and SAM-GFP-LNP study groups. CARS images were captured at 2884 cm^−1^ corresponding to the lipids (CH_2_) vibrational frequency. Cells treated with SAM-GFP-LNPs provide signal in the green fluorescence channel (571 ± 72 nm) indicating GFP expression. Scale bar corresponds to 10 µm.

For each study group, phosphate-buffered saline (PBS), control “empty” LNPs, or SAM-GFP-LNPs were passively delivered to adherent baby hamster kidney fibroblast cells (BHK-21), a cell line that is widely used for vaccine and recombinant protein production as well as viral transfection studies. The long-term fate of SAM-GFP-LNPs and empty LNPs in these cells was monitored by multimodal imaging techniques combining CARS, MPEF, and FLIM. Figure 1 shows the time-course of GFP expression intensity in SAM-GFP-LNP-treated BHK-21 cells (Fig. 1a), and the representative broadband CARS images overlaid with their fluorescence counterpart images of cells that were treated either with PBS (control), empty LNPs, or SAM-GFP-LNPs for a 24 h period (Fig. 1b). After the time course experiment, the viability of these cells was measured (Fig. S1) and then the cells were subsequently fixed in 4% paraformaldehyde (PFA) for other downstream measurements.

As shown in Fig. 1a, fluorescence signals indicating GFP expression in BHK-21 cells started to appear after 5 h of SAM-GFP-LNP treatment. Within 24 h, the GFP was markedly expressed in all SAM-GFP-LNP-treated cells, whereas the cells treated with empty LNPs or PBS did not exhibit any, as expected (Fig. 1b). SAM-GFP-LNPs and empty LNP-treated cells exhibited slightly delayed growth compared with the PBS-treated control cells, but they remained > 95% viable, as assessed by fluorescence microscopy of live cells after their exposure to propidium iodide (PI) and Hoechst 33342 dyes (Fig. S1). Given the strong diffusion of the GFP fluorescence signal visible in the cytosol and nuclei of the live cells at this time point, an increase in SAM expression was clearly evident at 24 h of treatment.

There were multiple bright regions visible in the CARS channel for the cells treated with empty LNPs or SAM-GFP-LNPs. These corresponded well with the strong CH_2_ vibrational response originating from the lipids observed in the CARS images (Figs. 1b and S2). The source of these lipidic CH_2_ signals for the cells treated with empty LNPs and SAM-GFP-LNPs could either emerge from the endogenous lipids or from exogeneous sources like LNPs. This necessitated further investigation into the source of the CH_2_ signals observed in the CARS images to determine if the endogenous lipids found in the native intracellular environment can be differentiated from the exogeneous lipids present in the LNPs. Utilizing the vibrational characteristics measured using HS-CARS, we analyzed the HS-CARS spectra thoroughly to identify if any changes on a pixel-to-pixel basis can be observed between the control, empty LNP, and SAM-GFP-LNP-treated cells. Since the broad nature of the HS-CARS spectra poses a challenge in resolving the center wavelength of the CH_2_ molecular vibrations, each spectral data was fitted to a mixture of Gaussian profiles (without any constraints), as explained in detail in the next section. The goal was to extract the fitted parameters and analyze them statistically to see if we can effectively separate and correlate both the spatial and spectral features following empty-LNP or SAM-GFP-LNP treatment.

### Development and evaluation of cell classification pipeline based on HS-CARS data

To demonstrate the discriminative power of HS-CARS features, the frequency response was modelled in terms of a Gaussian mixture with seven lineshapes for the spectra measured at each pixel^33,34^. Then, the model parameters were used as a high-level cell representation to distinguish between treated and untreated experimental groups. We posed the cell identification as a learn-based binary classification problem with noisy labels to overcome the lack of ground truth labels at a pixel-level and showed that the resulting decision boundary consistently predicts the cell category in multiple identification scenarios^35,36^.

**Figure 2:**
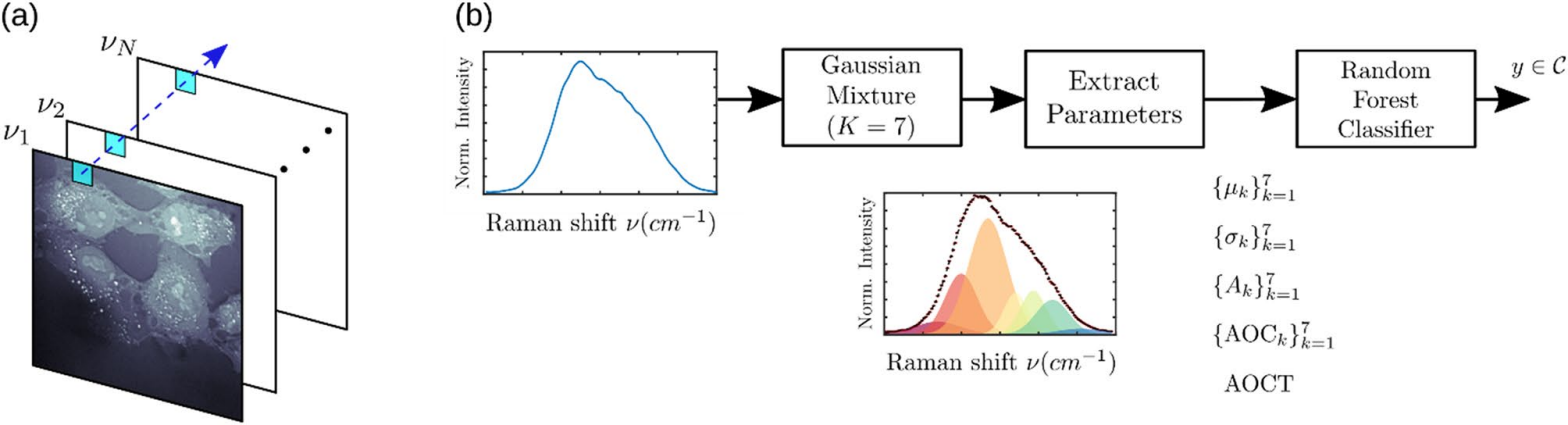
Cell classification pipeline. (**a**) Representative hyperspectral CARS dataset. (**b**) Cell classification pipeline: A random forest classifier was trained via the parameters of the Gaussian mixture model (GMM) components with the highest energy. The random forest ensemble was trained in a supervised fashion, learning a consistent decision boundary under a noisy label regime, i.e., by assuming all cells correspond to the same treatment group.

Detailed explanations regarding cell segmentation and lipid region identification are provided in the “Materials and methods” section. Briefly, the HS-CARS spectrum at a given pixel was divided into seven components uniformly covering the spectral wavenumber region from 2750 to 3100 cm^−1^, where the first three Gaussian components covered the lipid vibrational signatures, and the fourth and the fifth components covered the protein and nucleic acids vibrational signatures, respectively. As shown in Fig. 2, a cell classification pipeline was developed to identify cells (treated or not) with a high confidence through an ensemble learning algorithm using their fitted spectral properties (peak position, relative intensities, peak widths, and area under the curves). These spectral properties helped to identify the key features for predicting the cell category and to obtain a deeper understanding of the molecular origin of their chemical differences.

The normalized spectra obtained from each pixel were fitted to a combination of seven Gaussian lineshapes (Fig. 2b). A total of 29 parameters including amplitude, mean, standard deviation and area under the curve (AUC) for each of the seven Gaussian lineshapes were extracted from these fits. A supervised random forest classifier model was subsequently trained for the different cell treatment conditions because of its ability to avoid overfitting of unbalanced data and its ability to rank discriminative parameters. We focused on distinguishing between the following groups: (i) Control vs. LNP, (ii) Control vs. SAM-GFP-LNP, (iii) LNP vs. SAM-GFP-LNP, (iv) LNP vs. SAM-LNP, and (v) Control vs. LNP vs. SAM-GFP-LNP. The training was set up such that one classifier was developed for each scenario (total of 5 classifiers). We used 80% of such data points to train the random forest classifier, while the remaining 20% was used for testing purposes^37^. It is important to note that the main goal was to empirically show the discriminative representation ability based on the hyperspectral CARS response. The use of more refined classification algorithms, specialized methods of semi-supervised learning, and data pre-processing strategies are out of the scope of this analysis.

Classification performance was evaluated in terms of its Top-1 classification accuracy. Once the random forest classifier had been trained in a supervised fashion following a noisy labeling scheme, the cell identification consistency was evaluated by computing the ratio of pixels classified as affected by the treatment over the entire number of pixels. For that purpose, we randomly picked one high-resolution spectral map per group for each experiment and excluded these from the training process. Once the classifier was trained, these spectral maps were utilized to compute the prediction ratio for testing purposes. Top-1 classification accuracies of the 5 randomly initialized models are shown in Table 1. For each of the groups, > 90% mean prediction accuracy was obtained with a minimal standard deviation in all the groups, except for the Control vs. LNP vs. SAM-GFP-LNP group. The Top-1 classification accuracies for the individual experiments are shown in Table S1.

**Table 1:** Top-1 classification accuracy on full testing dataset.

	Top-1 classification accuracy (%)					
	Model initialization	Control vs. LNP	Control vs. SAM-LNP	Control vs. LNP vs. SAM-GFP-LNP	LNP vs. SAM-GFP-LNP	LNP vs. SAM-LNP
Full test dataset	Seed 0	91.11	93.8	87.31	92.27	90.33
	Seed 8	91.06	93.74	87.49	92.2	90.48
	Seed 16	91.05	93.68	87.49	92.09	90.42
	Seed 32	91.05	93.7	87.24	92.2	90.57
	Seed 42	91.19	93.68	87.41	92.22	90.37
	Mean	91.09	93.72	87.39	92.20	90.43
	Std	0.06	0.05	0.11	0.07	0.09

**Figure 3:**
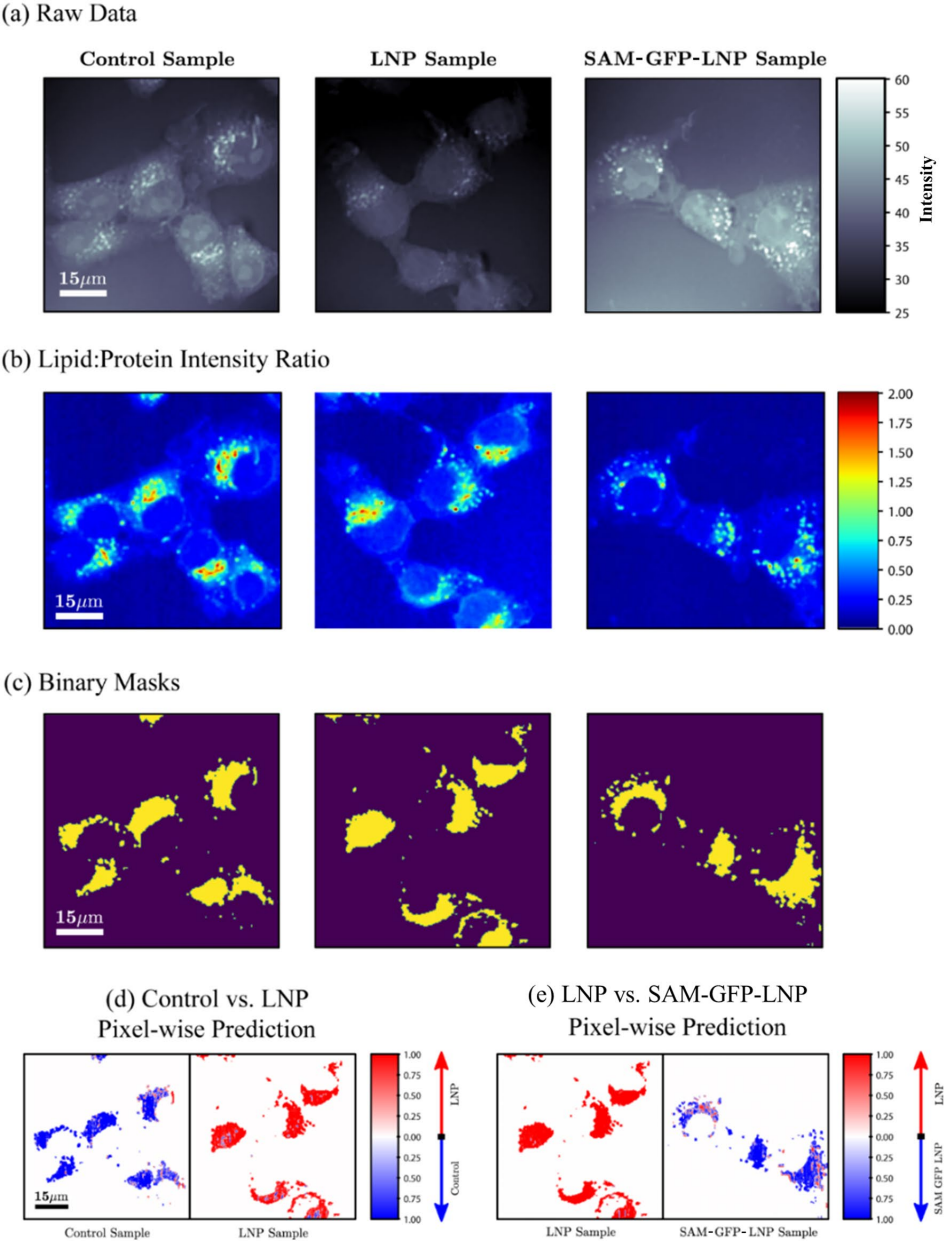
Cell classification based on HS-CARS data. (**a**) Raw HS-CARS images of control BHK-21 cells, cells treated with empty LNPs, and SAM-GFP-LNPs. (**b**) Representative images based on the lipid:protein ratio corresponding to different treatment conditions. (**c**) Binary masks to check the accuracy of classification metric based on the lipid:protein ratio. (**d**) and (**e**) Cell group predictions at each pixel are obtained under two classification scenarios: (i) Control vs. LNP and (ii) LNP vs. SAM-GFP-LNP. In both cases, most of the cell areas with high lipid:protein ratio are associated to the ground-truth cell group. The resulting probability maps show high-density areas, where the cell is most likely to be affected by the treatment. Scale bars correspond to 15 μm.

For the evaluation of the classification accuracy of the trained models, raw HS-CARS data with control cells, cells treated with empty LNPs and cells treated with SAM-GFP-LNPs were compared (Fig. 3a). In principle, cells treated with LNPs and SAM-GFP-LNPs should show stronger lipid signals than Control cells. The increased lipid signal in LNP and SAM-GFP-LNP groups could either be due to the increase in the lipid content following the addition of the LNP or could be due to changes in the packing of the acyl groups in the lipids. With the aim that the increased lipid signal could facilitate better classification of treated vs. untreated cells, the lipid:protein ratio was calculated to verify it as a metric for classification (Fig. 3b). The ‘yellow pixels’ show the pixels used for classification purposes based on the lipid:protein intensity ratio (Fig. 3c). Based on the lipid:protein ratio, the probability maps obtained from the control vs. LNP and LNP vs. SAM-GFP-LNP identification scenarios are shown in Fig. 3d and e, respectively.

### Determining changes in HS-CARS spectra associated with SAM-LNP uptake and interpretation of classification model performance

To understand the details of the molecular origin of the separation between different treatment groups, we first highlighted the mean frequencies that were extracted from the mixed Gaussian model and assigned them to different biomolecules present in the cell. The first 3 frequency bands centered around 2760 cm^−1^, 2830 cm^−1^, and 2860 cm^−1^ were attributed to the lipid vibrations originating predominantly from the CH_2_ stretching modes. The next two frequency bands centered around 2910 cm^−1^ and 2950 cm^−1^ originated from the protein and nucleic acid CH_3_ stretching vibrations, respectively. The variations of the peak frequencies of each of the HS-CARS spectral sub-bands over different groups are shown in Table 2. This information on the measured peak positions across different groups helps to understand the contribution of different GMM component parameters that affected the cell classification model performance.

**Table 2:** Peak positions of different HS-CARS spectral sub-bands.

Spectral sub-bands	Control	LNP	SAM-LNP	SAM-GFP-LNP
1	2767.89 (2.11)	2771.1 (0.55)	2766.15 (2.02)	2768.49 (2.16)
2	2823.98 (2.46)	2826.95 (3.30)	2827.11 (1.12)	2824.91 (2.34)
3	2856.42 (3.87)	2861.62 (3.07)	2860.33 (1.41)	2856.34 (1.89)
4	2910.18 (3.08)	2909.46 (2.53)	2905.84 (0.42)	2907.57 (2.36)
5	2949.25 (0.17)	2949.52 (1.09)	2946.91 (2.84)	2954.51 (0.60)
6	2997.05 (3.31)	2990.41 (1.43)	2989.24 (0.28)	2991.84 (0.72)
7	3045.66 (1.98)	3049.78 (2.91)	3051.87 (2.04)	3050.46 (2.53)

Values are shown as mean and standard deviation (Units: cm^-1^).

The top 10 features that influenced the model performance in correctly classifying the cells into different groups are shown in Table 3. The lipidic vibrations (first three sub-bands) played a crucial role for the separation between the Control group and the LNP group. Meanwhile, a separate set of these lipidic parameters (corresponding to the 2850 cm^−1^ peak) ranked higher for the classification between the Control and SAM-LNP groups. Since we observed a preponderance of lipidic regions in cells treated with LNPs and SAM-GFP-LNPs (Fig. 1), it is no surprise that the classification algorithm identified the lipid vibrations to contribute more towards a successful classification of Control and SAM-LNP groups as well. It is interesting to note that the same lipidic vibrations contributed towards classifying the cells treated with LNPs from those treated with SAM-LNPs. Furthermore, the first three ranked parameters of the classification algorithm were the same for LNP vs SAM-LNP and Control vs SAM-LNP groups.

**Table 3:** Random forest top 10 feature ranking.

	Control vs. LNP		Control vs. SAM-LNP		Control vs. LNP vs. SAM-GFP-LNP		LNP vs. SAM-GFP- LNP		LNP vs. SAM-LNP	
	Feature	Rank	Feature	Rank	Feature	Rank	Feature	Rank	Feature	Rank
1	amplitude1	0.16	AOCT	0.32	mu3	0.10	amplitude2	0.10	AOCT	0.23
2	sigma2	0.09	mu3	0.07	amplitude1	0.10	AOC2	0.10	mu3	0.09
3	sigma3	0.07	sigma3	0.06	AOCT	0.09	sigma3	0.08	sigma3	0.08
4	AOCT	0.06	mu1	0.05	sigma2	0.06	mu3	0.07	mu5	0.05
5	sigma5	0.06	mu4	0.04	sigma3	0.05	amplitude1	0.07	mu4	0.05
6	mu3	0.06	mu5	0.04	mu5	0.05	mu5	0.05	sigma4	0.05
7	mu5	0.06	sigma4	0.03	mu2	0.04	AOCT	0.04	mu2	0.04
8	AOC5	0.04	sigma2	0.03	sigma5	0.04	sigma2	0.04	sigma2	0.04
9	amplitude5	0.04	mu2	0.03	sigma4	0.04	sigma4	0.04	mu6	0.04
10	mu2	0.04	sigma5	0.03	mu4	0.04	amplitude6	0.03	sigma5	0.03

The parameter, total area under the curve (AOCT), contributed significantly towards LNP vs. SAM-LNP and Control vs. SAM-LNP classification. Not only did the AOCT have contributions from lipid, but it also reflected the total protein and nucleic acid represented in the spectra. Furthermore, it was noticed that the protein and the nucleic acid frequencies appeared later in the feature list in both these classification groups. Hence, the protein and the nucleic acid peaks might be relevant in identifying the cells treated with LNPs from those treated with SAM-LNPs. Based on these results, it seems that the HS-CARS measurements from the cells treated with the SAM-LNPs are sensitive to the mRNA components present in these treated samples. Our current spectral results could not identify any unique lipid features to differentiate exogenous lipids from endogenous lipids.

**Figure 4:**
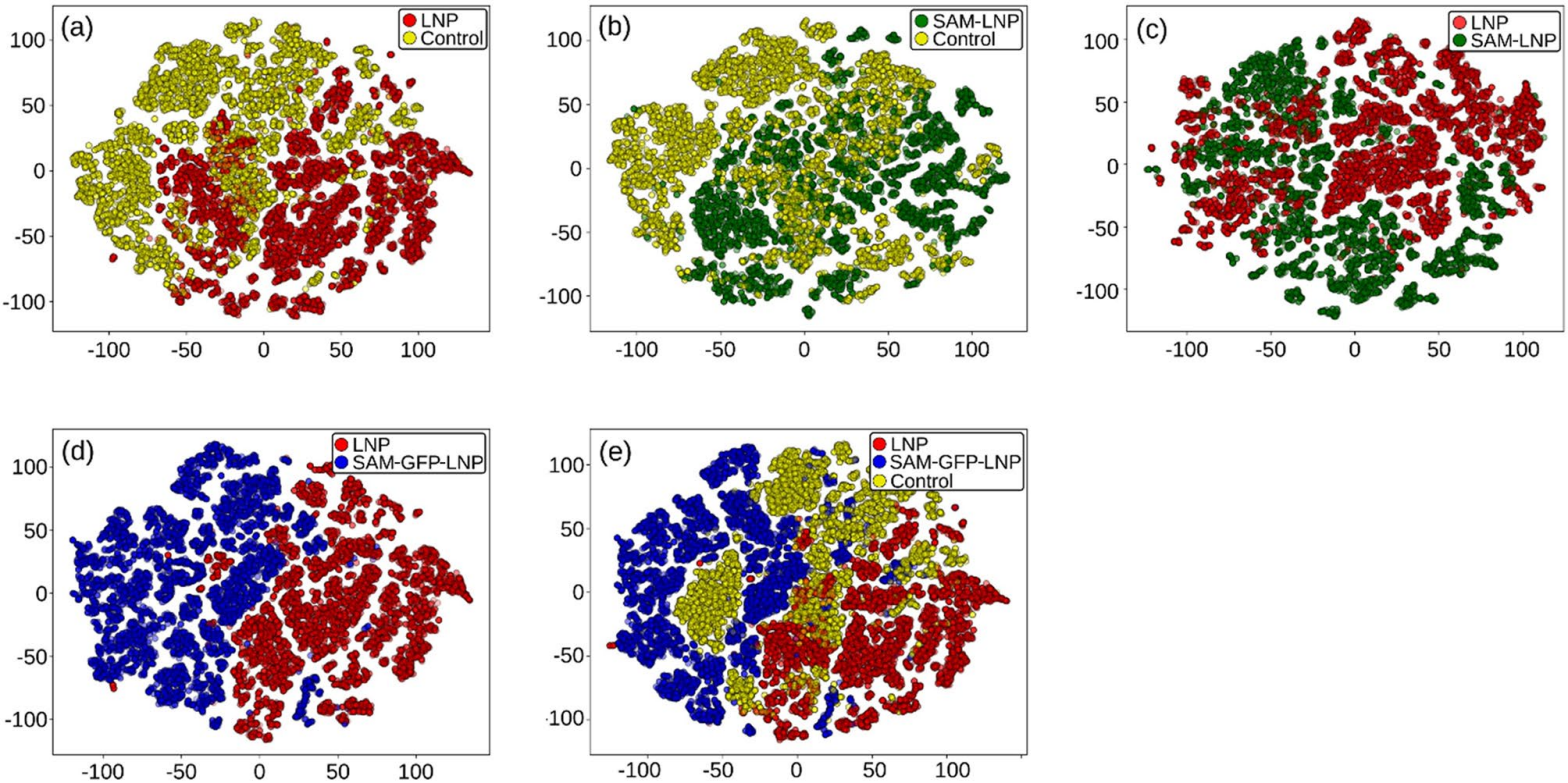
t-SNE plots. (**a**) Control vs. LNP group, (**b**) Control vs. SAM-LNP group, (**c**) LNP vs. SAM-LNP group, (**d**) LNP vs. SAM-GFP-LNP group and (**e**) Control vs. LNP vs. SAM-GFP-LNP group.

For the classification of the LNP vs. SAM-GFP-LNP groups, we would expect the nucleic acids and proteins to play a more crucial role as extra protein (GFP) is produced. However, contrary to our expectations, the first five important features indicate a trend towards lipidic vibrations. This might be because the added fluorescence of the GFP is affecting the spectral properties of the lipids more than the proteins or nucleic acids. The same observation holds true for the Control vs. LNP vs. SAM-GFP-LNP group as well. t-SNE plots of the distribution of the cells from different treatment conditions are shown in Fig. 4. t-SNE plots place similar cells together and different cells further apart. The plots in Fig. 4 display the successful distinction between Control and treated groups with different formulations. Thus, we demonstrated a random forest classification model that uses 29 extracted parameters from the HS-CARS images to distinguish between groups of cells.

### Classification of treatment groups and monitoring changes in the cellular microenvironment using FLIM

FLIM is an imaging technique sensitive to changes in the cellular microenvironment and is widely used for studying cellular metabolism. Fluorescence lifetime of endogenous fluorophores such as nicotinamide adenine dinucleotide (phosphate) (NAD(P)H) or flavin adenine dinucleotide (FAD) represent powerful biomarkers capable of providing information on the cellular microenvironmental changes in a non-destructive, label-free manner. In this study, we sought to examine whether NAD(P)H FLIM was capable of: (1) separating untreated cells from LNP- and SAM-GFP-LNP-treated cells, and (2) characterizing the cellular metabolic changes associated with the uptake of the vaccine. FLIM data of Control cells, cells treated with LNPs, and cells treated with SAM-GFP-LNPs were collected over a course of 6 h to track the cellular metabolic changes during the uptake and expression of SAM. Two binary classification problems (Control vs. LNP and Control vs. SAM-GFP-LNP) were considered to demonstrate the discriminative information provided by FLIM. Furthermore, features extracted spatially using the entire cell, only the nucleus, and only the cytoplasm, were compared. Classification results and the visualization of feature distributions localized the detectable effects of the SAM-GFP-LNP vaccine.

#### Classification of treated and untreated cells

The detailed algorithm used in the FLIM analysis pipeline is presented in the “Materials and methods” section. Briefly, individual cells were segmented and then either the nucleus, cytoplasm, or the entire cell region were masked to obtain unique parameters for cell treatment conditions (Fig. S3). Using CellProfiler, morphological features, co-location, and intensity were extracted according to each segmentation mask^38^. Thus, three possible feature sets per cell were used for downstream classification. For the Control vs. SAM-GFP-LNP dataset, 47 control cells and 100 SAM-GFP-LNP-treated cells were analyzed, while the Control vs. LNP dataset contained 53 control and 107 LNP-treated cells.

For each classification setting, two-thirds of the cells were randomly sampled for training and the remaining one-third were reserved for validation. Five hundred (500) random trials were performed for cross-validation and to calculate the mean validation accuracies. A random forest model was used to rank the most important features for classification according to the Gini impurity metric^39^. We empirically found that a random forest classifier was the best-performing model for our single-cell classification tasks.

Mean classification accuracy results for both the Control vs. SAM-GFP-LNP and Control vs. LNP datasets using each feature set are presented in Table 4. The best classification results were obtained using the cytoplasm features for each experiment, while nuclei-based features were clearly the lowest-performing. Since roughly two-thirds of the cells belonged to the treated group in each experiment, a naïve random guess would have provided about 66% classification accuracy. This suggested that nuclei features showed weak differences between the control and treated experimental groups, while the cytoplasm showed evident changes. Top-1 classification accuracy was higher for the Control vs. SAM-GFP-LNP group (85.6%) compared to the Control vs. LNP group (80.4%).

**Table 4:** FLIM Top-1 classification accuracy.

Comparison groups	Top-1 classification accuracy (%)		
	Nucleus	Cytoplasm	Cell
Control vs. SAM-GFP-LNP	70.5	**85.6**	84.0
Control vs. LNP	71.5	**80.4**	79.4

The mean validation accuracy across 500 random splits for each experiment and set of features is shown. The best performing features for each experiment are shown in bold.

**Figure 5:**
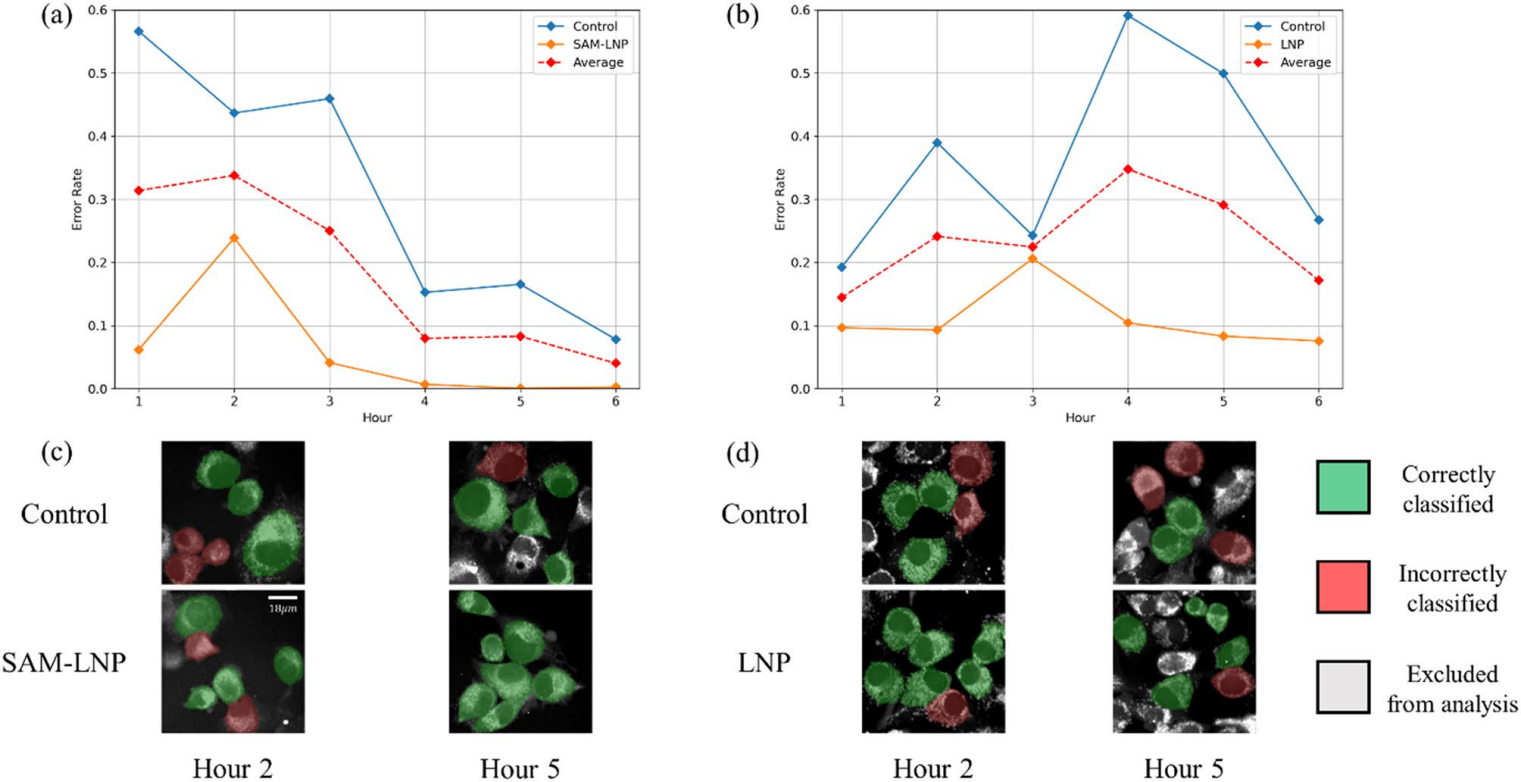
Validation error rate against time. (**a**,**c**) Control vs. SAM-GFP-LNP and (**b**,**d**) Control vs. LNP groups. The red dashed line provides the average error rate for each group in each experiment. Cells that were correctly and incorrectly classified are shown in green and red color, respectively.

Further, the validation accuracy for classifying cells at each time-point was examined. Figure 5 shows the mean error rate (complement of the classification accuracy) for classifying cells at each of the six hours for the FLIM data. Notably, SAM-GFP-LNP-treated cells were easier to separate from Control cells as time passed due to the GFP expression associated with the SAM, while the classification of Control against empty LNPs did not correlate with time. An apparent improvement in classification between SAM-GFP-LNP and Control cells at the 4^th^ hour was observed, which also holds for later time periods as well. This agrees with Fig. 1a, where GFP expression was detected after 4 h. No such trend emerged when classifying between empty LNP and Control groups. The images in the bottom panel of Fig. 5 illustrate the improvement in the classification as a function of time in SAM-GFP-LNP-treated cells compared to LNP-treated cells. This demonstrates that our classification model is sensitive to detect SAM-GFP expression.

#### Correlation of FLIM features with treatment condition

During FLIM data analysis, it was found that using just the top five CellProfiler features, according to the random forest feature importance, yielded the best performing models for each biological experiment. For the Control vs. SAM-GFP-LNP treatment scenario, the top three features were the Rank-weighted Colocalization (RWC)^40^ and Costes correlation^41^ coefficients between the bound NAD(P)H fraction (β_1_) and mean fluorescence lifetime (τ_m_), and the minimum intensity of the fluorescence lifetime of bound NAD(P)H (τ_1_). The Control vs. LNP experiment had top features that also included the Costes correlation and the minimum intensity of the τ_1_ channel, in addition to the minimum edge intensity of the τ_1_ channel.

In brief, the Costes correlation between two image channels or masks measures the fraction of image energy that lies above a statistically significant threshold. Thus, pixels below this threshold are roughly uncorrelated. The Costes correlation coefficient for a given cell lies between zero and one. If the coefficient is close to zero, the co-localization is dominated by noise (lower SNR); conversely, if the coefficient is close to one, SNR is higher (see the Supporting Information for more detailed explanations of Costes and RWC correlation coefficients).

**Figure 6:**
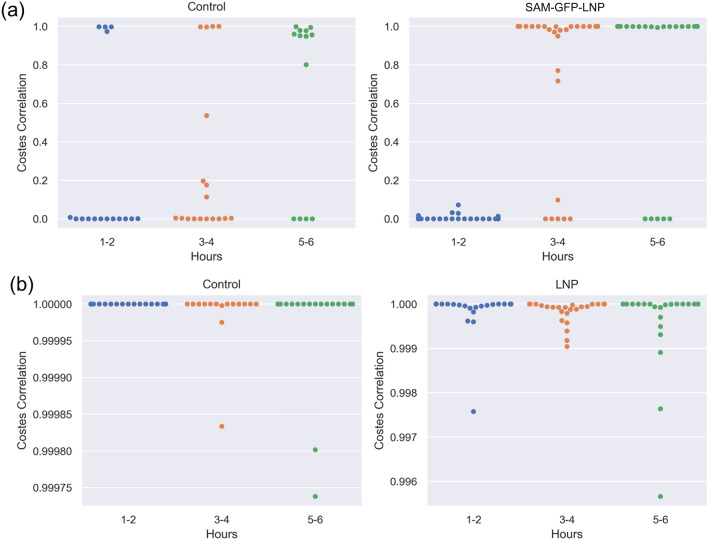
Feature distributions of the Costes correlation coefficient between β_1_ and τ_m_ channels within the cell cytoplasm. The distribution of Costes correlation coefficient values between β_1_ and τ_m_ channels over time for the (**a**) Control vs. SAM-GFP-LNP data and (**b**) Control vs. empty LNP. This feature was the top feature for models trained for both collected FLIM datasets.

Figure 6 demonstrates how the Costes correlation coefficient between β_1_ and τ_m_ channels varies over time between Control and LNP treatments, and Control and SAM-GFP-LNP treatments. After the 3–4 h time point, the SAM-GFP-LNP data becomes strongly correlated between β_1_ and τ_m_ channels, while the corresponding Control data does not display such a change. This is consistent with our findings from the 4th hour in Fig. 5 where these two groups become more differentiated. The observed trend in Fig. 6a, suggests that the SNR for this experiment is relatively low. However, a metabolic change might be expressed within the SAM-GFP-LNP treated cells that strengthens the correlation between the β_1_ and τ_m_ over time. No such phenomenon is seen for the cells treated with empty LNPs and their corresponding control data. The Costes correlation for this group of classification consistently remains close to one (Fig. 6b). This suggests the data for the Control vs. LNP experiments has higher SNR, while the discriminative power comes from the treated group having *lower* correlations than the control group. This is counter to the trend seen with the SAM-GFP-LNP treated cells (Fig. 6a). A direct comparison between LNP vs. SAM-GFP-LNP was not performed due to changes in experimental conditions between the two runs.

Therefore, the FLIM results suggest that the correlation between the NAD(P)H population and the mean fluorescence lifetime becomes stronger over time in the cells treated with SAM-GFP-LNPs, in the cytoplasmic regions of the cell, when compared to Control and LNP-treated cells. These results indicate that the presence of SAM and associated GFP expression during the first 6 h post-treatment led to increased functional activity in the cells treated with SAM-GFP-LNPs. Further studies are needed to gain a mechanistic understanding of the extent of functional changes occurring in the cellular microenvironment following SAM-GFP expression.

## Conclusions

In summary, we used a multimodal optical imaging platform with HS-CARS and multiphoton-excited FLIM imaging capabilities to study the cellular uptake of a SAM-GFP-LNP vaccine in vitro using BHK-21 cells. By integrating HS-CARS and MPEF (intensity and lifetime) data, we demonstrated that it takes around five hours for the SAM to be expressed in BHK-21 cells. The time taken by cells to express SAM varies, but it reaches equilibrium by 24 h after vaccine treatment. Using learn-based classification models, we were able to accurately identify cells that were treated vs. untreated. Accurate classification results based on HS-CARS imaging were obtained using the lipid:protein ratio. Using our cell classification pipeline, it was possible to classify cells treated with empty LNPs and SAM-LNPs with ~ 90% Top-1 classification accuracy and the contributions of different HS-CARS spectral sub-bands to the model performance were determined. Additionally, FLIM results showed stronger correlation between the bound NAD(P)H population and the mean fluorescence lifetime during the SAM expression in cells treated with the SAM-GFP-LNP vaccine than in untreated cells, indicating increased functional activity within the cytoplasm during SAM uptake and its expression. These results demonstrate the strength of multimodal, multiphoton, and spectroscopic imaging techniques to obtain functional and spatial information about vaccine uptake and expression in cells. Further studies using labelled as well as label-free detection techniques providing sub-cellular spatial resolutions and biochemical information are warranted to investigate targeted vaccine delivery in different types of cellular and tissue microenvironments.

## Materials and methods

### Reagents

All chemicals and analytical lab reagents were ACS ((≥ 95%) of higher purity grade. Water was LC/MS Grade (≥ 99.9%).

### Cell cultures

An adherent Syrian golden hamster (*Mesocricetus auratus*) kidney fibroblast cell line (BHK-21, clone 13, ATCC #CCL-10) was used in this study. Cells were cultured in disposable Corning™ 75 cm^2^ vented-cap cell culture treated flasks in phenol red-free Gibco™ Dulbecco's Modification of Eagle's Medium (DMEM) (Thermo Fisher Scientific (TFS), #21063029) containing 25 mM HEPES, 4 mM l-Glutamine, 25 mM d-glucose (dextrose) and supplemented with 5% heat-inactivated HyClone™ Characterized Fetal Bovine Serum (FBS) (Cytiva, #SH30071.03) as well as 1% Gibco™ Antibiotic–Antimycotic (penicillin/streptomycin/amphotericin B) solution (TFS, #15240062). Cells were maintained inside a humidified incubator with 5% CO_2_ and 21% O_2_ conditions at 37 °C until they reached 80% confluency. Cells were routinely passaged and sub-cultured (split) by trypsinization using phenol red-free 0.25% Trypsin–EDTA solution, incubation time of 3 min, and neutralization with 10% FBS-containing DMEM. A 0.5–1 ml volume of harvested cells was resuspended in 1.5–1 ml of phenol red-free GibcoTM 1X TrypLE™ Select Enzyme (pH 7.0–7.4) cell dissociation reagent (TFS, Cat #12563029) in triplicates. The total and viable cell numbers, cell diameter, and viability (%) were estimated using a Beckman Coulter Vi-CELL XR Automated Cell Viability Analyzer under default mammalian cell type settings.

### Cell treatment with experimental compounds and fixation by cross-linking

For intracellular drug delivery and subsequent imaging studies, the cells were seeded in sterile 35 mm glass-bottom poly-D-lysine coated imaging dishes with 14 mm well size and #0 coverslip (Cellvis, Mountain View, CA, #D35-14-0-N; MatTek, Ashland, MA, #P35GC-0–14-C) at varying cell densities (for pilot experiments) or at one fixed density of 0.75 × 10^6^ cells/ml. The cells were treated at 24 h post-attachment to the substrate with 3.3 µL PBS (vehicle control), empty LNP, SAM-LNP, or SAM-GFP-LNP (0.3 µg in a final 2 mL complete cell culture medium volume) compounds in technical triplicates for up to 24 h.

A stock of methanol-free EM grade 16% paraformaldehyde (PFA) aqueous solution (Electron Microscopy Sciences, Hatfield, PA) was diluted with HyClone™ 0.1 µM filter-sterilized Phosphate Buffered Saline (PBS) without calcium and magnesium (pH 7.0 – 7.2) (Cytiva, Marlborough, MA) to obtain fresh 4% PFA solution. Complete cell culture media was aspirated, and the cells were rinsed with PBS twice prior to adding 2 ml of 4% PFA. After 20 min incubation at RT in the dark, the PFA was removed, and the cells were washed with PBS three times. The cells were imaged immediately or were stored at 4 °C in 1 × PBS no longer than a week. In exploratory studies, the cells were spiked with 500 µl of 4% PFA for 2 min, then fixed in 8% PFA for 20 min and quenched with 0.1 M Glycine.

### Qualitative assessment of cell viability by fluorescence microscopy

The ReadyProbes™ Cell Viability Imaging Kit (Blue/Red) was used to evaluate cell viability 24 h post-drug treatment. The kit contains propidium iodide (PI) (Ex/Em = 535/617 nm) and Hoechst 33342 (Ex/Em = 360/460 nm) dyes. Two drops of a PI/ Hoechst 33342 fluorophore solution were added to each 35 mm dish with live cells. After 10 min of cell incubation at 37 °C, the emission of blue (Hoechst 33342), green (GFP), and red (PI) fluorescence signal was simultaneously captured by a ZEISS Axio Observer microscope using DAPI/FITC/TexasRed channels at low or intermediate power magnification using 5×, 10×, or 40× objectives.

### Assessment of cell vitality by AlamarBlue assay

Cell vitality and metabolic functions were evaluated by an AlamarBlue assay following protocols recommended by the manufacturer.

### Hyperspectral coherent anti-Stokes Raman scattering (HS-CARS) and multiphoton excitation fluorescence (MPEF) microscopy

The multimodal optical microscopy system comprising of CARS and MPEF was built on a commercial upright BX51 Olympus microscope. A dual output 80 MHz femtosecond (fs) laser source (Chameleon Discovery, Coherent) was employed to generate the laser excitation pulses. The tunable laser beam (660 nm to 1300 nm) was tuned to 800 nm and used as the pump beam. The 1040 nm fixed wavelength output was used as the Stokes beam for CARS microscopy to excite C-H bond vibrations at 2884 cm^−1^. The simultaneous absorption of 800 nm and 1040 nm laser beams contributed to the MPEF of green fluorescent protein (GFP) encoded by the SAM-GFP-LNP molecules.

We combined the pump and the Stokes beams spatially and temporally through a dichroic beam splitter (Di02-980, Semrock) and a motorized translational stage (X-LSM050A-KX13A, Zabor Technologies Inc.), respectively. For HS-CARS imaging, the overlapped beams were chirped using two SF-10 glass rods, each 150 mm long. HS-CARS was performed using a spectral focusing method by tuning the optical delay between the two chirped beams. A motorized delay stage was employed to sweep a distance of 1.2 mm with a 5 µm step size while collecting a single-wavelength CARS spectrum at each time delay. The spectral range covered was between 2750 and 3100 cm^−1^.

A 2D galvo mirror scanning system was used to raster scan the laser beams across the sample. After dipping the 40× water immersion objective with a numerical aperture of 0.8 (LUMPLFLN, Olympus) into the imaging dishes containing cells, the CARS signal was collected in a transmission geometry with a photomultiplier tube (PMT) (H7422-40, Hamamatsu) and a bandpass filter (650/13 nm, FF01-650/13/25, Semrock) to reject the excitation pulses. The MPEF signal was captured in the epi direction with another PMT (H7422-40, Hamamatsu) and a set of dichroic mirrors and filters were used to detect the fluorescence signal from GFP in the range of 571 ± 72 nm. For femtosecond (fs) CARS of cells and MPEF microscopy of cells treated with SAM-GFP-LNPs, the fs pump and Stokes beams were used directly without chirping them through the glass rods. We used ~ 10 mW of Stokes and pump beam power at the sample and covered the microscope with a black curtain to reduce ambient light from leaking into the PMTs.

The galvo mirrors were set to a step voltage of 0.003 V and the resultant 400 × 400 pixel image covered a field-of-view (FOV) of 75 × 75 µm^2^. A pixel dwell time of 10 µs was used, which corresponded to a total acquisition time of 1.6 s per image. Two pre-amplifiers (PMT-4V3, Advanced Research Instruments Corp.) and a current–voltage converter were used to pre-amplify the CARS and MPEF signals before they were acquired by the data acquisition system (PCIe-6351, National Instruments). A home-built LabVIEW program was utilized to scan the laser beams and acquire the spatially co-registered HS-CARS and MPEF images.

### Fluorescence lifetime imaging microscopy (FLIM)

The same dual output 80 MHz fs laser source (Chameleon Discovery, Coherent) generated the laser excitation pulse to detect the metabolic changes in the cells using a custom-built FLIM system. For two-photon NAD(P)H excitation in live cells, the beam was tuned to a wavelength of 750 nm and sent to an inverted microscope equipped with a 40×, 0.75 NA objective to focus the light on the cells. Two-photon-excited NAD(P)H fluorescence intensity and lifetime signals were acquired in epi geometry. A band pass filter centered at 450 ± 53 nm (FF01-451/106-25, Semrock) was used to filter out the NAD(P)H signals from other cellular signals, and a PMT (H7421-40, Hamamatsu) detected the fluorescence emission. The imaging dishes with live cells were placed on a motorized XY piezo stage which was accompanied by a heating stage set to a temperature of 37 °C.

We used ~ 25 mW of laser power at the sample and two galvo mirrors to scan a FOV of 90 × 90 µm^2^ covering 512 × 512 pixels. To avoid photobleaching, each FOV was assessed only once. To study the intermediate and short-term changes in the cellular microenvironment associated with vaccine uptake in cells, FLIM data was collected over a period of 6 h post cell treatment. At the beginning of each hour, one FOV was obtained from a dish containing control (PBS-treated) naïve cells, followed by imaging dishes with experimentally treated cells. A total of 4–5 FOVs were obtained at different imaging dish locations, with a ~ 10 min interval between subsequent FOVs. After each imaging session, the dish was returned to a portable humidified incubator. At the end of each hour, the control sample was imaged once again (to assess the potential changes in cellular metabolism caused by the imaging process itself at given environmental conditions). For the subsequent imaging session, a new set of three dishes with cells was used. This procedure was repeated up to 6 h post-treatment.

### Data modeling analysis

#### HS-CARS data fit

At each pixel, HS-CARS data was characterized as a Gaussian mixture model (GMM) comprised of seven components uniformly covering from 2750 to 3100 cm^−1^. Based on this configuration, the first three Gaussian components covered the lipid vibrational signatures, while the fourth and fifth components were attributed to the protein and nucleic acids vibrational signatures. The data fitting process was performed via a nonlinear least squares optimization problem^42^, providing an estimate of the mean, standard deviation (SD), and amplitude of each of the seven components. Based on the key observation that the spectral peak, predominantly located around 2850 cm^−1^, shifts depending on the status of cell treatment, we hypothesized that this was caused by changes on the second, third, and fourth Gaussian components. More precisely, the peak position changes were explained in terms of the parameters of its three closest Gaussian components.

#### Cell refinement

To differentiate between exogeneous and endogenous lipids, we focused mainly on the regions with high lipid signal intensity. To filter out those regions, only those cell areas with a high lipid:protein ratio were preserved and subsequently used for the classification. This cell refinement was achieved by computing the ratio between lipid and protein intensities, which correspond to the energy of their sub-bands (2750 cm^−1^ to 2890 cm^−1^ for lipids and 2890 cm^−1^ to 2990 cm^−1^ for proteins and nucleic acids). Once the intensity ratio was obtained from all cell regions, a relative threshold based on Otsu’s method^43^ was applied to keep only those highly activated areas.

#### Cell identification

Next, the cell group identification problem was casted as an *N-way* classification task with noisy labels. Specifically, we were interested in distinguishing between the following groups: (i) *Control vs. LNP*, (ii) *Control vs. SAM-LNP*, (iii) *LNP vs. SAM-LNP*, and (iv) *Control vs. LNP vs. SAM-GFP-LNP*. Given the lack of ground-truth labels at a pixel level, the concept of *random classification noise* was used and assumed that all refined cells in a group corresponded to the same category.

The amplitude, mean, and standard deviation of the seven GMM components were used as high-level representations of the spectral response. Based on this compact representation, the best decision boundary was extracted from data by training a random forest classifier with 100 estimators. The use of random forests allowed one not only to obtain a competitive classification performance in a supervised fashion, but also to rank the feature importance^44^. The latter provided insight about how meaningful each Gaussian component and their corresponding spectral sub-bands were towards identifying the cell group.

### Single-cell analysis pipeline

#### Cell segmentation (FLIM)

Each cell was individually segmented using the routine Cellpose algorithm^45^. The whole cells in each FOV were manually selected by excluding any cells that were cut off at the edge of an image. These segmentations were further enriched by manually outlining masks for each cell nucleus. This allowed us to generate separate feature maps for each cell by looking only at the nucleus, cytoplasm, or the entire cell. Figure S3 shows the example segmentation masks for one FOV. For the Control vs. SAM-GFP-LNP dataset, 47 control cells and 100 SAM-GFP-LNP-treated cells were analyzed, while the Control vs. LNP dataset contained 53 control and 107 LNP-treated cells. Since two datasets were obtained on different days, so different control samples were imaged concurrently with each treatment group.

#### Feature extraction

Based on the segmentation mask for each cell, Intensity, morphological, intensity distribution, and colocalization/correlation features were extracted using CellProfiler and any features that contained NaN values or had zero variance across all cells were removed. In total, this provided approximately 320 features for the classification and characterization of each cell. Furthermore, it computed these features using the cell-level, nuclei, and cytoplasm segmentation masks and reported results for each of these three choices of feature sets.

### Supplementary Information

Supplementary Information.

## Data Availability

The data that support the findings of this study are available from the corresponding author upon reasonable request and through collaborative investigations.
