# Supplementary material for: Probing delivery of a lipid nanoparticle encapsulated self-amplifying mRNA vaccine using coherent Raman microscopy and multiphoton imaging
Source: Sci Rep. 2024 Feb 22;14:4348. doi: 10.1038/s41598-024-54697-3 (PMC10884293; doi:10.1038/s41598-024-54697-3)
Supplement: Supplementary file 1 — Supplementary Information. [file 41598_2024_54697_MOESM1_ESM.docx]

**Probing Delivery of a Lipid Nanoparticle Encapsulated Self-Amplifying mRNA Vaccine using Coherent Raman Microscopy and Multiphoton Imaging**

Supporting Information

Kajari Bera^1,2,#^, Renán A. Rojas-Gómez^1,3,#^, Prabuddha Mukherjee^1,2^, Corey E. Snyder^1,3^, Edita Aksamitiene^1,2^, Aneesh Alex^1,4^, Darold R. Spillman Jr^1,2^, Marina Marjanovic^1,2,5,6^, Ahmed Shabana^7^, Russell Johnson^7^, Steve R. Hood^1,8^, Stephen A. Boppart^1,2,3,5,6,9*^

^1^GSK Center for Optical Molecular Imaging, University of Illinois Urbana-Champaign, Urbana, IL, USA

^2^Beckman Institute for Advanced Science and Technology, University of Illinois Urbana-Champaign, Urbana, IL, USA

^3^Department of Electrical and Computer Engineering, University of Illinois Urbana-Champaign, Urbana, IL, USA

^4^In vitro/In vivo Translation, Research, GlaxoSmithKline, Collegeville, PA, USA

^5^Department of Bioengineering, University of Illinois Urbana-Champaign, Urbana, IL, USA

^6^Carle Illinois College of Medicine, University of Illinois Urbana-Champaign, Urbana, IL, USA

^7^GSK Vaccines, Rockville Center for Vaccines Research, Rockville, MD, USA

^8^In vitro/In vivo Translation, Research, GlaxoSmithKline, Stevenage, UK
^9^Cancer Center at Illinois, University of Illinois Urbana-Champaign, Urbana, IL, USA

*To whom correspondence should be addressed. [boppart@illinois.edu](mailto:boppart@illinois.edu), (217) 244-7479

**BHK-21 cell viability after 24 h of treatment:**

SAM-GFP-LNP- and LNP-treated cells exhibited slightly delayed growth compared to the PBS-treated control cells, but they were >95% viable, as assessed by fluorescence microscopy of live cells after their exposure to propidium iodide (PI) and Hoechst 33342 (**Figure S1**) dye. PI is a membrane impermeant dye that is generally excluded from viable cells. It binds to double stranded DNA by intercalating between base pairs. Cell membrane-permeable Hoechst 33342 stains both live and dead cell nuclei.


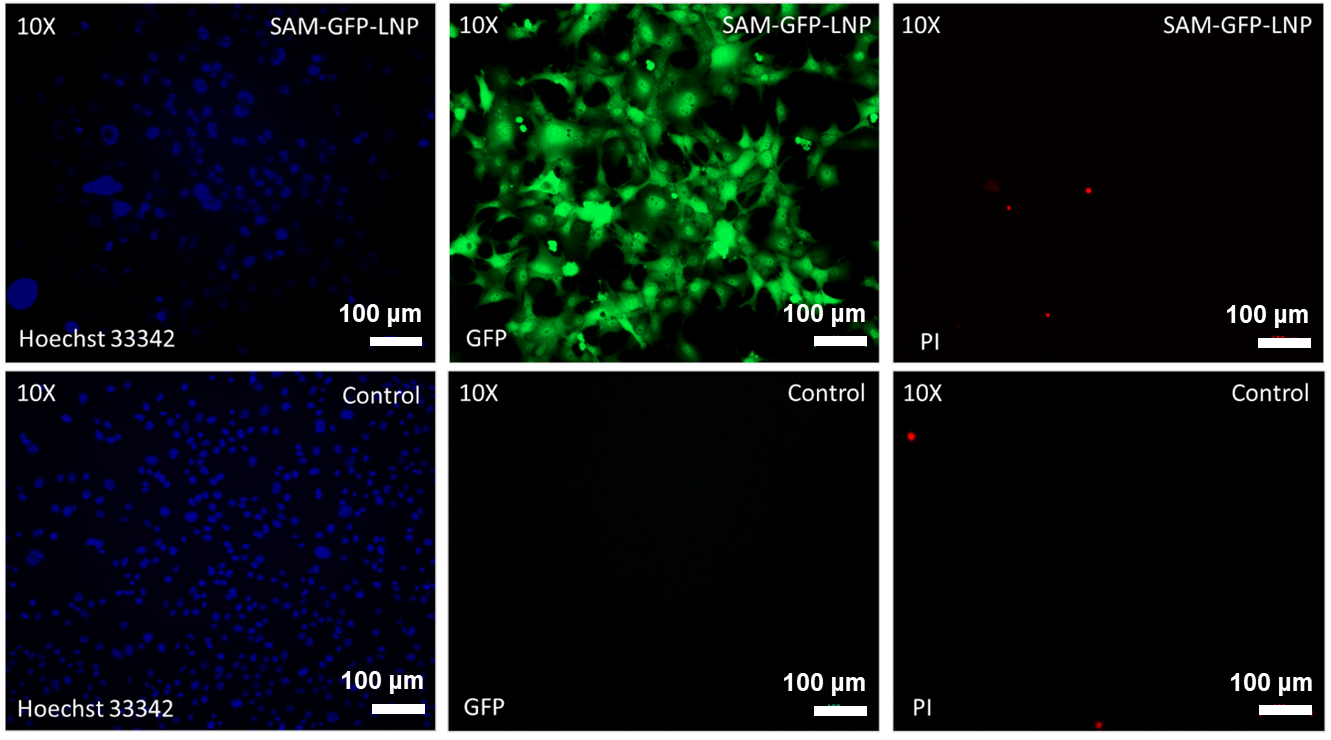


Figure S1: **BHK-21 cell viability 24 h after treatment with PBS (control) or SAM-GFP-LNPs.** Total cell number is indicated by both live and dead cell nuclei stained with Hoechst 33342 (left column), while dead propidium iodide (PI) stain-positive cell nuclei are shown in red (right column). Green fluorescent protein signal (GFP) confirming the SAM expression is shown in the middle column. Scale bars in 10x magnification images are 100 μm.

**Intracellular lipid droplets visualized by simultaneous Oil Red O staining:**

Stock Oil Red O (ORO) solution was prepared by dissolving 0.5 g Oil Red O in 100 mL isopropanol (IPA) at 37 °C followed by passing through the 0.22 µm pore filter. A working solution was prepared by diluting 6 ml of ORO stock solution with 4 ml of MBG H_2_O followed by additional passing through the 0.05 µm size pore syringe filter with 1.0 µm GF prefilter (Tisch Scientific, Clever, OH, Cat # SF18256). Fixed cells were incubated with 60% IPA for 5 min. IPA was discarded, and cells were covered with 2 ml of working ORO solution for 15 min. Once ORO was aspirated, cells were washed 5 times with MBG H_2_O. Hematoxylin solution was added dropwise and kept for 1 min prior to final wash with MBG H_2_O 4 times, and the addition of 2 mL PBS. In other cases, 8%-PFA fixed cells were first stained with LipidSpot™ 610 lipid droplet stain (LS610) (Biotium, Fremont, CA) (Ex/Em = 592/638 nm) for 10 min prior to ORO staining. Alternatively, cells were first stained with LS610, then fixed in 8% PFA and stained with ORO. Stained lipids were visualized by brightfield and fluorescence microscopy using an oil immersion objective at 63× magnification. Pseudocolor green was used to visualize LS610 signals merged against ORO.

There were multiple bright spots in images that exhibited strong CH_2_ signal in the CARS channel, which corresponds to lipids. These spots typically coincided with the location of lipid droplets, as confirmed by an independent imaging analysis of BHK-21 cells treated with ORO (a lipid-soluble lysochrome with a maximum absorption at 518 nm used for the staining of neutral lipids (triglycerides and diacylglycerols)) and/or membrane-permeant LipidSpot™ 610 lipid droplet stains (Biotium) (**Figure S2**).

**
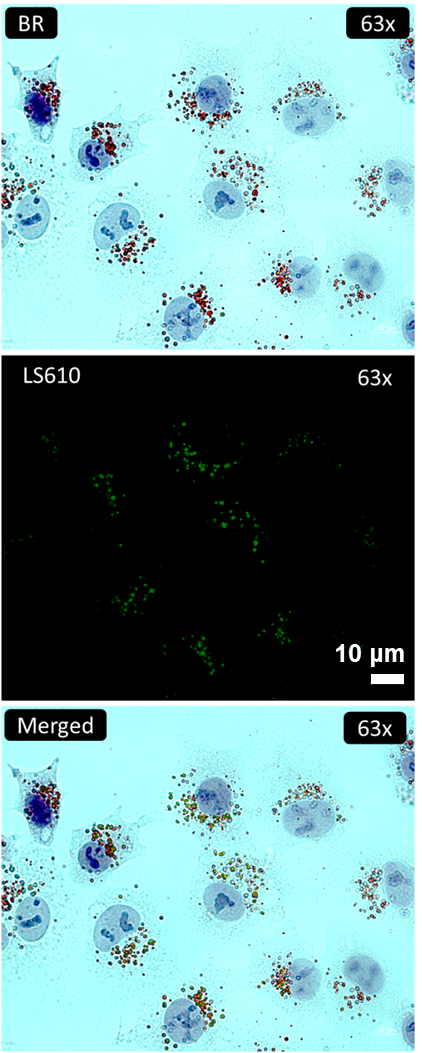
**

Figure S2: **Lipid droplet identification.** Lipid droplets visualized by simultaneous Oil Red O (red color in brightfield, blue - nuclei) and LipidSpot™ 610 (Biotium, green fluorescence pseudocolor) lipid droplet staining in BHK-21 cells. Single-channel and merged representative images taken at 63x magnification are shown. Scale bar corresponds to 10 μm.

**Model performance for individual experiments:**

**Table S1:** Top-1 classification accuracy on single experiments. Values are shown as mean and standard deviation.

| Experiment no. | **Top-1 Classification Accuracy (%)** | | | | |
| --- | --- | --- | --- | --- | --- |
|  | **Control vs. LNP** | **Control vs. SAM LNP** | **Control vs. LNP vs. SAM-GFP-LNP** | **LNP vs. SAM-GFP-LNP** | **LNP vs. SAM-LNP** |
| 1 | 90.19 (0.15) | - | 86.21 (0.17) | 88.71 (0.21) | - |
| 2 | 91.50 (0.19) | - | 84.14 (0.18) | 90.20 (0.27) | - |
| 3 | 85.16 (0.46) | 91.67 (0.23) | 85.06 (0.36) | 90.77 (0.08) | 83.12 (0.29) |
| 4 | **97.13 (0.16)** | 87.23 (0.17) | 89.42 (0.27) | 93.28 (0.07) | 85 (0.17) |
| 5 | 89.84 (0.14) | **97.57 (0.04)** | 88.25 (0.21) | 94.25 (0.12) | **96.28 (0.04)** |
| 6 | 90.77 (0.23) | 91.01 (0.18) | **91.17 (0.16)** | **96.76 (0.12)** | 87.41 (0.5) |

**Cell segmentation for FLIM data:**

We segmented each cell individually using the Cellpose algorithm [45]. Whole cells in each field-of-view (FOV) were manually selected by excluding any cells that were cut off at the edge of an image. We further enriched these segmentations by manually outlining masks for each cell nucleus. This allowed us to generate separate feature maps for each cell by looking only at the nucleus, cytoplasm, or the entire cell. **Figure S3** shows a representative segmentation mask for a representative FOV.

**
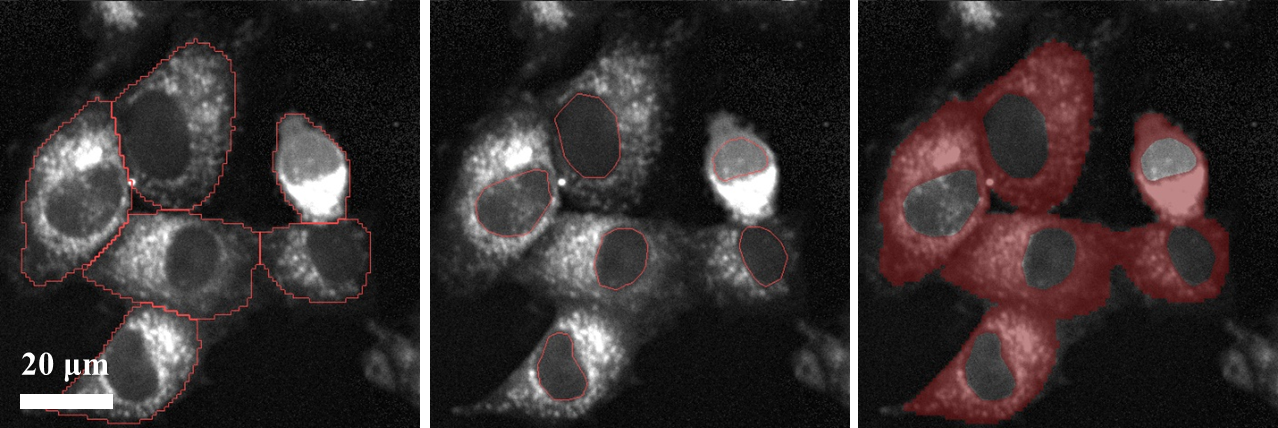
**

Figure S3: **Segmentation of entire cells (***left***), their nuclei (***middle***), or cytosol (***right***) regions**. These masks were used to generate single-cell features at the “cell”, “nuclei”, and “cytoplasm” levels.

**t-SNE evaluation: Spatial arrangement and inter-group distance of t-SNE embedding**

This section details the spatial arrangement and inter-group distance of t-SNE plots shown in Figure 4 of the main text. Each treatment group's two-dimensional embedding was computed using t-SNE with perplexity 16, early exaggeration 12, and PCA initialization. Embeddings were generated through 1,000 iterations, revealing the spatial distribution and relative positioning of different treatment groups.

To quantify the separation between cell groups in a low-dimensional space, we calculated the centroid (average point) for each group and measured their Euclidean distances. This complements the t-SNE visualization, where similar cells cluster and dissimilar ones are pulled apart. Table S2 shows the calculated centroids and distances in the t-SNE embedding space. Notably, the centroids showed consistent spacing across groups, further emphasizing the significant distinction between control and treated cells, especially with different formulations. This finding aligns with our random forest classification model, which successfully distinguishes cell groups based on various extracted parameters from HS-CARS images.

**Table S2:** **Embedding proximity and distribution across treatment groups.**

| **Experiment** | **Euclidean Distance** | **t-SNE centroids ([x,y])** | | | |
| --- | --- | --- | --- | --- | --- |
|  |  | **Control** | **LNP** | **SAM-LNP** | **SAM-GFP-LNP** |
| **LNP vs. SAM-GFP-LNP** | 93.36 | - | [48.09, -11.96] | - | [-42.65, 10] |
| **LNP vs. SAM-LNP** | 42.65 | - | [9.61, 17.02] | [-10.45, -20.62] | - |
| **Control vs. SAM-LNP** | 45.8 | [-14.51, -14.4] | - | [18.43, -17.42] | - |
| **Control vs. LNP** | 74.25 |  |  | - |  |
| **Control vs. LNP vs. SAM-GFP-LNP** | *Control vs. LNP*: 71.45  *Control vs. SAM-GFP-LNP*: 61.97  *LNP vs. SAM-GFP-LNP*: 109.38 | [7.73, 24.56] | [50.48, -32.69] | - | [-51.59, 6.63] |

Though embedding distances in t-SNE support the high classification accuracy of our method, it is important to highlight that t-SNE projects high-dimensional data into lower dimensions using a non-linear mapping approach. This means the global structure of the real data might not be perfectly captured in the t-SNE representation. However, despite this caveat, t-SNE remains a valuable tool for both visualization and clustering, as evidenced by its successful applications in various settings.
